# Supplementary material for: Awareness of Antimicrobial Resistance and Associated Factors among Layer Poultry Farmers in Zambia: Implications for Surveillance and Antimicrobial Stewardship Programs
Source: Antibiotics (Basel). 2022 Mar 14;11(3):383. doi: 10.3390/antibiotics11030383 (PMC8944564; doi:10.3390/antibiotics11030383)
Supplement: Supplementary file 1 [file antibiotics-11-00383-s001.zip › antibiotics-1631561-supplementary.pdf]

antibiotics

MDPI

Supplementary material:

Table S1. Questionnaire.

**1. GENERAL INFORMATION**	
**Question**	
**Date of survey**	…………../………../……………… (DD/MM/YYYY)
Name of interviewer	
Contact details of interviewer	
**2. DEMOGRAPHICS**	
Sex of respondent	1. Male<br>2. Female
Marital status	1. Married<br>2. Single<br>3. Never married<br>4. Widow/widower<br>5. Divorced
Religion of respondent	1. Christianity<br>2. Muslim<br>3. Hinduism<br>4. Others (specify)
Nationality of respondent	1. Zambian<br>2. Non-Zambian
Education level of respondent	1. No education<br>2. Primary<br>3. Secondary<br>4. Tertiary
**3. EPIDEMIOLOGICAL DETAILS**	
Farm identification code	
Veterinary camp	
Source of water	1. Borehole<br>2. Well<br>3. Stream/river<br>4. Pipe-borne<br>5. Others (specify)
Production category/type	1. Broiler<br>2. **Layer**<br>3. Traditional
Farm category	1. Small [1-1000]<br>2. Medium[1001-10000]<br>3. Commercial [above 10000]
Geographical location of Farm/Collection point (Coordinates in degree decimals e.g. lat -14.43807 long 28.45251)	Lat:<br>Long:
Province	
District	
Source of chickens (name of hatchery if known)	
**4. AMU and AMR Details**	
Use of antimicrobials in layer production	1. Yes<br>2. No
Use of antibiotics in the current stock of chickens	1. Yes

	2. No
If yes, list the antimicrobials used (in order of quantity usage)	
Do you keep records of antibiotics used in the poultry?	1. Yes<br>2. No
Route of administration of antibiotics	1. Oral<br>2. Topical<br>3. Others (specify)
Do antibiotics manage to treat infections in chickens?	1. Yes<br>2. No<br>3. Sometimes
Do you also use traditional medicines to treat infections in chickens?	1. Yes<br>2. No
Common diseases/condition and corresponding antimicrobials used on the birds being sampled	Disease/Condition Antimicrobial
Use of chicken droppings at the farm	
Source of antimicrobials used	1. Agrovet shop<br>2. Veterinary Officer<br>3. General Pharmacy<br>4. Fellow farmers<br>5. Not accessed<br>6. Other (specify)
Who do you consult the use of antibiotics?	1. Veterinary officers<br>2. Agro-vet personnel<br>3. General pharmacy personnel<br>4. Other farmers<br>5. Others (specify)
Use of a prescription to access antimicrobials	1. Yes<br>2. No
Do you consult veterinary officers on the use of antibiotics?	1. Yes<br>2. No
Knowledge on observation of withdrawal periods<br>E.g. *"Do you sell eggs and meat of animals while they are under treatment?" followed by "If not, who long do you wait after the end of the treatment to sell eggs? meat?"*	1. Yes<br>2. No<br>3. Not sure
Treatment of market ready birds	1. Yes<br>2. No
Use of antibiotics for treating infections	1. Yes<br>2. No<br>3. Not sure
Use of Antimicrobials to prevent diseases	1. Yes<br>2. No<br>3. Not sure
Use of Antimicrobials to improve egg production	1. Yes<br>2. No

	3. Not sure
Use of feed additives	1. Yes<br>2. No
Type of feed used	1. Commercial<br>2. Self-formulated
Does the feed have antimicrobials	1. Yes<br>2. No<br>3. Not sure
If yes, provide the name of the antimicrobial/s	______________________
How long is the medicated feed given to the flock?	
Who administers antibiotics?	1. Farm owner<br>2. Farm worker<br>3. Others (specify)
Where do you store antibiotics for use in the farm?	1. Farmhouse shelf/ cupboard<br>2. Refrigerator in the residence<br>3. Refrigerator in the farm<br>4. Others (specify)
Are you aware of antimicrobial resistance?	1. Yes<br>2. No
If yes, what's your source of AMR information?	1. Veterinary officers<br>2. Agro-vet personnel<br>3. General pharmacy personnel<br>4. Other farmers<br>5. Others (specify)
Explain antimicrobial resistance	
**5. BIOSECURITY DETAILS**	
Protective Equipment put on by workers	1. Gum boots<br>2. Work suits<br>3. Overalls<br>4. Others (specify)
Is the poultry environment fenced?	1. Yes<br>2. No
Are poultries restricted to personnel only?	1. Yes<br>2. No
List other animal species on the same farm (e.g. ducks, geese, guinea fowl, goats, cattle, pigs, fish)	
House for sick birds	1. Yes<br>2. No
Type of floor in poultry	1. Concrete<br>2. Soil<br>3. Others (specify)
Disinfectants availability (for spraying the pens, foot baths)	1. Yes<br>2. No
How are dead birds gotten rid of?	1. Burnt<br>2. Buried<br>3. Cooked for beds<br>4. Eaten by workers<br>5. Others (specify)

Presence of rats in chicken houses	1. Yes<br>2. No
How are rats gotten rid of?	1. Poisoned<br>2. Eaten by rats<br>3. Others (specify)
Type of chicken houses	1. Cages<br>2. No cages
Farm check on biosecurity measures implemented	……………………………………………………………………<br>……………………………………
